# Consistent seasonal flexibility of the gut and its regions across wild populations of a winter-quiescent fish

**DOI:** 10.1098/rsos.231975

**Published:** 2024-03-20

**Authors:** Timothy J. Fernandes, Hugo Li, Brian J. Shuter, Bailey C. McMeans

**Affiliations:** ^1^ Department of Biology, University of Toronto Mississauga, 3359 Mississauga Road, Mississauga, Ontario L5L 1C6, Canada; ^2^ Department of Ecology and Evolutionary Biology, University of Toronto, 27 King's College Circle, Toronto, Ontario M5S 1A1, Canada; ^3^ Aquatic Research and Development Section, Ontario Ministry of Natural Resources and Forestry, 300 Water Street, Peterborough, Ontario K9J 8M5, Canada

**Keywords:** phenotypic flexibility, seasonality, ecophysiology, aquatic ecosystems, Centrarchidae

## Abstract

Seasonality in north-temperate environments imposes drastic temperature and resource variations that shape the seasonal ecophysiology of resident organisms. A better understanding of an organism’s capacity to flexibly respond to this drastic seasonal variation may reveal important mechanisms for tolerating or responding to environmental variation introduced by global change. In fishes, the digestive system is both the interface between resource and energy acquisition and one of the most expensive organ systems to maintain. However, little evidence describing the capacity for seasonal flexibility in the digestive tract of wild northern fishes exists. Here, we investigated phenotypic flexibility in the size of the gastrointestinal (GI) tract across three northern populations of a winter-dormant warm-water fish, pumpkinseed sunfish (*Lepomis gibbosus*). In all populations, pumpkinseed exhibited pronounced structural flexibility in the GI tract, aligned with winter and the timing of reproduction. The dry mass of the GI increased by 1.3- to nearly 2.5-fold in the early spring. The pyloric caeca demonstrated the greatest capacity for flexibility, increasing by up to 3.7-fold prior to reproduction. In all populations, minimum dry GI mass was consistently achieved during winter and mid-summer. This capacity for gut flexibility may represent a novel mechanism for facilitating rapid adaptive responses (e.g. metabolic plasticity) to future environmental change.

## 1. Introduction

At northern latitudes, seasonal transitions represent predictable but often dramatic shifts in abiotic conditions, especially temperature, food availability, oxygen and light. As ectotherms, fishes are extremely sensitive to changes in environmental temperatures and exhibit a suite of physiological and ecological responses to seasonal variations in abiotic conditions [1–3]. To efficiently acquire energy in seasonal environments, fish may alter the form and function of their digestive machinery, stimulated by changes in the quantity or quality of diet items [3–7]. Such digestive flexibility has been documented extensively in the laboratory across taxa; however, our understanding of the capacity for flexibility in the gut of wild northern fishes, especially in response to seasonally dynamic energetic demands, is limited [3,8].

Modifications to the size and structure of the gut facilitate efficient digestion and allow organisms to tailor the costs of digestion to available resource conditions and energetic requirements [9]. Though increases in gut size and capacity may improve energy acquisition and digestive efficiency [4,8,10], larger, more complex guts also carry implicitly higher metabolic costs [11,12]. Indeed, digestion can consume more energy than exhaustive exercise in some fishes [13], with the maintenance of the digestive tract representing nearly 25% of an individual’s aerobic metabolism [14]. Seasonal decreases in gut size could therefore function as an energy conservation strategy during periods of reduced resource accessibility, while increased gut size may facilitate improved digestive capacity when resources are abundant [12,15–17]. As global change in the North continues to introduce unpredictable environmental variation [18], the ability to flexibly modulate the size, capacity and energetic cost associated with the gut may become increasingly impactful for combating seasonal resource and energetic uncertainty. However, our understanding of what mediates gut flexibility in fishes is largely based on evidence collected from south-temperate and tropical species.

Here, we used northern populations of pumpkinseed sunfish (*Lepomis gibbosus*) to investigate intra- and inter-seasonal flexibility in the structural mass of the gastrointestinal (GI) tract. Pumpkinseed sunfish are generally inactive during the winter and rely on both stored and newly acquired energy for reproduction during the spring [19]. Thus, there is a high demand for digestive capacity immediately after winter dormancy to rapidly fuel spring reproduction. We hypothesized that if the digestive machinery is flexibly regulated to both improve energy assimilation and minimize energetic costs across seasons, then the GI tract will decrease in size during inactive winter months, when both foraging and energetic demands are probably low, followed by increases in size prior to reproduction in the spring when both foraging and energetic demands are probably high. We also predicted that higher temperatures (i.e. increased metabolic efficiency) in the presence of food and lower temperatures (i.e. decreased metabolic efficiency) in the absence of food could promote both a decline in gut mass as a mechanism for maximizing net energy gain and minimizing net energy loss, respectively.

## 2. Methods

### 2.1. Fish sampling and dissections

Pumpkinseed were sampled approximately biweekly from January to August 2021 (table 1) across three private ponds (less than 2 ha) in Southern Ontario (Pond A, Pond B and Pond C; table 2). Pumpkinseed sunfish were the top predatory fish species and represented the most abundant fish across all ponds. Fish from Pond C exhibited a smaller maximum length than both Pond A and Pond B (table 1), probably representing a stunted population as described previously in Ontario [20]. However, our aim was to explore whether the GI was seasonally flexible in all three study populations, regardless of potentially divergent life histories.

**Table 1.** Sampling schedule and sample sizes as counts of females captured between 60 and 110 mm that were dissected and used for analysis of GI flexibility. All sample periods with less than three fish were excluded from analyses but presented in figures.

sample	date range	pond A	pond B	pond C	total
1	16 to 22 Jan 2021	10	4	5	19
2	5 to 11 Feb 2021	5	4	2	11
3	19 to 21 Feb 2021	5	5	0	10
4	5 to 8 Mar 2021	9	5	0	19
5	17 to 19 Mar 2021	12	5	0	17
6	3 to 9 Apr 2021	9	0	5	14
7	15 to 20 Apr 2021	5	5	0	10
8	4 to 8 May 2021	7	4	4	15
9	18 to 20 May 2021	11	7	6	23
10	6 to 8 Jun 2021	8	5	0	13
11	22 to 24 Jun 2021	3	1	2	6
12	7 to 8 Jul 2021	4	0	1	5
13	16 Jul 2021 (single day)	0	5	2	7
14	1 Aug 2021 (single day)	3	3	4	10
total		91	53	31	175

**Table 2.** Morphological and ecological description of the study sites sampled for pumpkinseed sunfish.

pond	maximum depth (m)	surface area (ha)	maximum total length^a^ (mm)	fish community^b^
pond A	2.1	0.29	182	Iowa darter, northern redbelly dace and pumpkinseed
pond B	5.5	1.1	201	brown bullhead, fathead minnow, northern redbelly dace and pumpkinseed
pond C	5.5	0.56	125	pumpkinseed

^a^ Across all samples and sexes.

^b^ Brown bullhead (*Ameiurus nebulosus*), fathead minnow (*Pimephales promelas*), pumpkinseed (*Lepomis gibbosus*), Iowa darter (*Etheostoma exile*) and northern redbelly dace (*Chrosomus eos*).

Fish were captured via angling and clover-leaf fish traps (1.2 × 1.2 × 0.3 m) baited with approximately 50 ml of chicken-flavoured kibble in an enclosed bait container preventing consumption of kibble by trapped fish. Captured fish were euthanized via percussive stunning followed by pithing. Euthanized fish were immediately placed on ice and transported to the Koffler Scientific Reserve for dissection. Within 6 h of euthanasia, fish were measured (±0.1 cm), weighed (±0.001 g) and dissected for collection of wet organ masses (±0.001 g). Gonadosomatic index (GSI) and gut fullness index (GFI) were calculated as follows:


$$GSI=\frac{G_{m}}{gRWT}\times 100,$$

$$GFI=\frac{{SC}_{m}}{gRWT}\times 100,$$

where *gRWT* is the wet mass of the gutted fish carcass lacking viscera and gonads (±0.001 g), $G_{m}$ is the wet mass of the gonads (±0.001 g) and ${SC}_{m}$ is the wet mass of stomach contents calculated as the wet mass of the GI tract including stomach contents less the wet mass of the GI tract without stomach contents (±0.001 g). All dissected organs were then placed in pre-labelled microcentrifuge tubes and immediately frozen at −20°C. Mature female fish between 60 and 110 mm in total length were processed for GI tract analyses, as energy demands associated with reproductive development are intensified in females, and this was the most abundant size class across all ponds and sampling periods. GI tract samples were moved to the University of Toronto Mississauga campus for processing, where each was dissected into three regions: stomach (including the oesophagus), pyloric caeca and intestine. Cuts were made at common constriction points anterior and posterior to the pyloric caeca. Any remaining contents were cleared and discarded, and each region was placed on a pre-weighed and labelled aluminium pan. The wet weight of each region was recorded (±0.0001 g), followed by drying at 60°C until a constant mass was achieved (72 h; e.g. [8]).

### 2.2. Statistical analyses

All statistical analyses were conducted in R v. 4.3.1 [21]. We used analysis of covariance (ANCOVA) to detect seasonal differences in sample masses within each pond [22]. ANCOVA included either dry GI tract mass or dry region mass as a response variable, the sampling period (table 1) as the independent variable and gutted body mass as the continuous covariate. All continuous variables were natural-log transformed prior to analysis. If sample periods exhibited unequal slopes or model residuals were non-normally distributed, we analysed body-size-corrected region masses using Kruskal–Wallis non-parametric analysis of variance (ANOVA). Tukey’s pairwise contrasts were conducted to detect pairwise differences in mean values between sample periods [23]. When presenting the magnitude of differences in text, we reported estimated marginal means ±s.e [24]. All data and code have been made publicly available as electronic supplementary material.

## 3. Results

### 3.1. Seasonal flexibility of the gastrointestinal tract and its regions

**Figure 1.**
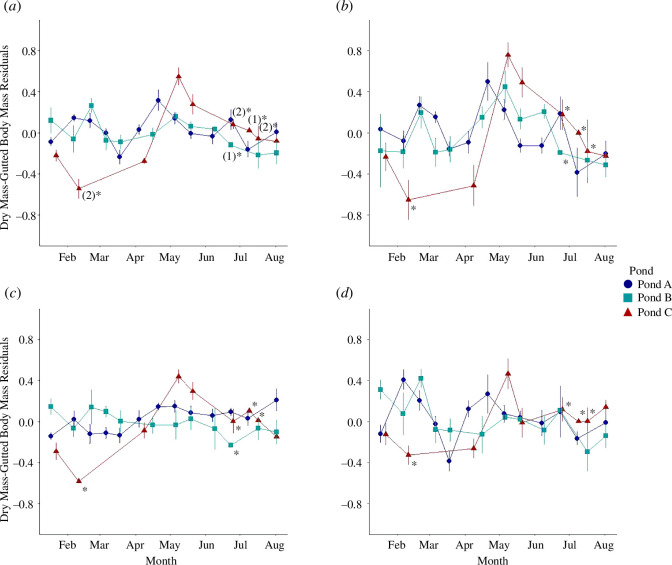
Dry mass of the entire GI tract (*a*), pyloric caeca (*b*), stomach and oesophagus (*c*) and intestine (*d*) in female pumpkinseed sunfish between 60 and 110 mm (Pond A: blue; Pond B: teal; Pond C: red). Points represent the mean residual values for each sampling event, extracted from the relationship between the natural-log-transformed GI region and the natural-log-transformed gutted body mass of each population. Asterisks indicate points that were excluded from analyses owing to the low sample sizes reported in brackets. Error bars indicate ±1 s.e.

**Figure 2.**
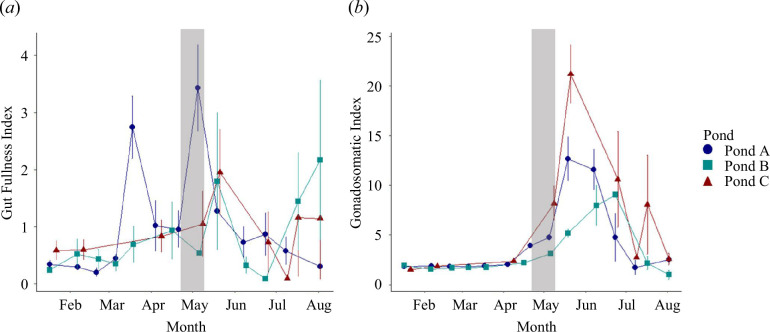
Seasonal patterning of foraging (*a*) and reproductive allocation to the gonads (*b*) in female pumpkinseed sunfish between 60 and 110 mm. The shaded region depicts the timing of peak dry GI mass across all populations (Pond A: blue; Pond B: teal; Pond C: red). Error bars indicate ±1 s.e.

The GI tract exhibited strong seasonal variation in all three populations sampled (figure 1*a*; Pond A: *F*_12, 76_ = 4.60, *p* < 0.001; Pond B: *F*_10, 39_ = 3.03, *p* < 0.01; Pond C: *F*_4, 18_ = 26.56, *p* < 0.0001). In Pond C, the GI tract exhibited the highest level of variation, more than doubling in total dry mass from early April (24.7 ± 2.3 mg; *n* = 5) to early May (59.0 ± 5.0 mg; *n* = 4; Tukey’s pairwise contrast, *t* = 8.52, *p* < 0.001). Similarly, in Pond A, GI mass nearly doubled from mid-March (28.4 ± 1.5 mg; *n* = 12) to mid-April (50.3 ± 4.1 mg; *n* = 5; Tukey’s pairwise contrast, *t* = 3.48, *p* < 0.05). Though fish from Pond B increased GI mass modestly (1.3-fold) from late winter to early spring, spring peaks in GI mass occurred during a period of increasing relative gonad mass and immediately prior to foraging peaks across all populations (figure 2*a,b*). GI mass then declined from spring to summer by at least 25% in all populations. Peaks in dry mass were also present in mid-winter in Pond A and Pond B.

Relative to other gut regions, the seasonal patterning of the pyloric caeca was the most congruent across populations. Also, the pyloric caeca exhibited the largest degree of seasonal flexibility (Pond A: *F*_12, 77_ = 4.61, *p* < 0.001; Pond B: *F*_10, 39_ = 2.70, *p* < 0.05; Pond C: *F*_4, 18_ = 12.20, *p* < 0.001), increasing 1.9- to 3.7-fold from winter to early spring across all populations (figure 1*b*). Maximum pyloric caeca mass occurred between late April and late May across all populations, while minima occurred either during the winter or mid-summer months.

Similarly, the dry mass of the stomach peaked between late April and late May in Pond A and Pond C (figure 1*c*; Pond A: Kruskal–Wallis χ^2^ = 27.32, d.f. = 12, *p* < 0.01; Pond C: Kruskal–Wallis χ^2^ = 23.71, d.f. = 8, *p* < 0.01); Pond B exhibited a slight peak during the winter and a slow decline in mass through spring and into the summer that was not seasonally significant (Kruskal–Wallis χ^2^ = 7.59, d.f. = 11, *p* > 0.1).

Intestinal mass peaked most strongly during the early winter in Pond A (Pond A: *F*_12, 77_ = 3.71, *p* < 0.001), decreasing by more than half from early February (18.7 ± 2.4 mg) to mid-March (8.5 ± 0.7 mg; Tukey’s pairwise contrast, *t* = 5.21, *p* < 0.01), followed by a secondary peak in mid-April (16.6 ± 2.1 mg; Tukey’s pairwise contrast, *t* = 4.31, *p* < 0.01). Intestine mass then declined consistently from early spring to summer. Pond B mirrored these patterns, though fish exhibited a milder increase in the early spring (figure 1*d*; Pond B: *F*_10, 39_ = 2.27, *p* < 0.05). In Pond C, intestine mass was significantly reduced during winter, increasing sharply by 2.3-fold in the early spring (Pond C: *F*_4, 18_ = 8.96, *p* < 0.001; Tukey’s pairwise contrast, *t* = 5.43, *p* < 0.001), and then decreasing through the summer similarly to Ponds A and B.

## 4. Discussion

Here, we investigated the capacity for seasonal flexibility in the GI tract of pumpkinseed sunfish. Across three populations, fish demonstrated significant seasonal variability in the mass of their digestive machinery, increasing the dry mass of the entire GI by between 1.3- and nearly 2.5-fold from winter to early spring. Surprisingly, dry GI mass increased during mid-winter in two of the sampled populations, followed by peaks immediately preceding reproduction. Significant increases in dry GI mass were rapid, occurring across 2-week periods, with the largest observed changes occurring in less than a month. Following early-spring peaks, GI mass declined through the summer in all populations regardless of increased foraging later in the year. Thus, pumpkinseeds do not appear to regulate the size of their GI tract in response to feeding alone. However, the timing and magnitude of structural GI modulation varied across populations. Most notably, the smallest-bodied (potentially stunted) population (Pond C) exhibited the greatest capacity for flexibility with strong increases in dry gut mass prior to reproduction, while fish from Pond A and Pond B exhibited secondary peaks in dry gut mass during winter. Future work should explore whether population-level differences in life history (e.g. stunted versus large-bodied) consistently influence seasonal flexibility in the gut (via divergent seasonal energetic demands, resource availability, or seasonal activity patterns), especially in pumpkinseed sunfish, where stunted populations are known to invest more energy into developing reproductive tissue [20]. In spite of population-specific idiosyncrasies, all populations exhibited the following consistent patterns: (i) increases in overall GI mass, especially pyloric caeca, prior to reproduction; (ii) reductions in overall GI mass, especially pyloric caeca, during progression through summer; and (iii) the pyloric caeca represented the most flexible and consistently patterned GI region. Though beyond the scope of this work, identifying the mechanisms driving consistent flexibility in the pyloric caeca and population-specific differences in GI flexibility remains a fruitful avenue for future work. The ability to adjust the structure, capacity and cost associated with operating and maintaining the gut may represent a novel mechanism for meeting energetic demands following environmental change in northern fishes.

Extensive laboratory considerations in mammals, reptiles, birds and, more recently, fishes have demonstrated the taxonomically generalized nature of gut flexibility [16,25–30]. Though rare, data generated from wild fish populations show strong concordance with patterns observed here. Wild bull trout (*Salvelinus confluentus*) exhibited strong intra-annual GI flexibility, where fish increased the size of their GI tracts by nearly threefold to capitalize on an annual pulse of salmon eggs [8]. Interestingly, the degree of gut flexibility shown in bull trout was similar to the degree demonstrated by pumpkinseed here. Though the observed GI augmentation in bull trout was associated with increased foraging, fish were also actively developing reproductive tissue between the two sampling periods [8]. Thus, modulation of the GI tract may be a more general response to fuelling reproduction in fish than is currently appreciated, especially in highly seasonal environments.

In the laboratory, fishes exhibit rapid (e.g. hourly to daily) gut flexibility in response to diet shifts and starvation [6,31]. However, little is currently understood about gut flexibility in wild fish populations and digestive responses to changing energy demands. Work in marine and freshwater fish has suggested that foraging contributes most strongly to the regulation of GI mass [4,6,7]. Yet, pumpkinseed exhibited structural growth in the GI tract before, potentially in anticipation of, peaks in foraging, with increases in GI mass matching initial gonad development (figure 2*b*). Increased foraging only elicited a structural response in the gut prior to gonad development. Thus, the GI tract appears capable of responding to the dynamic seasonal energy demands imposed by reproduction, with increased GI mass during reproductive development probably reflecting increased digestive capacity. Interestingly, two of the three studied populations exhibited increases in the size of the digestive tract during winter months when foraging was low. Such increases in gut size during low-temperature periods may facilitate the maintenance of digestive capacity when suboptimal temperatures would otherwise inhibit digestion [32,33], potentially fuelling reproductive preparation in the liver that can occur during the winter in pumpkinseed [34]. Similarly, maintaining digestive capacity during the winter may allow fish to use low levels of feeding to combat the depletion of energy reserves, assuming sufficient resources are available [35]. As warm summer temperatures drive increased digestive efficiency, the costs of maintaining and operating a large gut are probably no longer necessary given the temperature-dependent increases in digestive capacity [36,37]. Such modulation of the gut and its regions may be a broad mechanism for achieving seasonal metabolic plasticity in northern fishes, from centrarchids to salmonids [1,38].

Though each region of the GI tract considered here generally followed the same seasonal pattern, the pyloric caeca exhibited notably greater structural flexibility than the stomach and intestine. Relative to other organs and gut regions, the pyloric caeca in bull trout also demonstrated the greatest degree of augmentation [8]. Further, flexibility in the pyloric caeca of Atlantic cod (*Gadus morhua*) was most critical for maximizing post-starvation growth [39,40]. Yet, much of the work on digestive plasticity in fishes has traditionally focused on the intestine [3,6,10,41]. Though caecal tissue appears to be the most structurally flexible, this distinction may represent divergent mechanisms of digestive modulation. Increased enzymatic activity in the intestine not reflected in its mass may be more advantageous for improved digestive efficiency [42], while the size and capacity of caeca may be critical for improving digestive surface area and storing consumed food to supply a constant flow of digesta to the intestine [9,43]. Regardless of historical attention, the pyloric caeca again emerge as an integral yet under-studied region of the fish gut [43]. Though the specific mechanism driving the observed differences between the gut regions is unknown, all regions of the GI exhibit the capacity for structural modulation in response to seasonal foraging and energy dynamics, within and across populations.

Altogether, these results suggest that the size and structure of the GI tract exhibit the capacity for flexibility within and between seasons across wild populations of a common north-temperate fish. This capacity for seasonal flexibility may be central to the regulation of both digestive capacity and metabolic maintenance costs. Indeed, populations or individuals with a greater capacity for seasonal flexibility in their digestive machinery may be better positioned to capitalize on unpredictable resource and temperature conditions. However, further work is needed to assess the explicit response of the GI tract to dynamic energetic demands and foraging in fishes. With GI maintenance alone occupying 25% of an individual’s oxygen consumption [14], increasing the size of the gut by upwards of twofold probably results in direct consequences on whole-animal metabolism, reducing the available scope for aerobic processes like activity and growth [13]. To further investigate how structural flexibility in the gut may shape fish energetics and populations, we must better understand the drivers and metabolic costs of upregulation and downregulation in the gut (e.g. how does the aerobic cost of digestion and gut maintenance change following structural changes in the GI tract?). To what extent does flexibility in the gut represent a mechanism for driving metabolic plasticity (e.g. metabolic depression [37])? This work also raises questions regarding the role of the GI tract in shaping seasonal biology across species. Is the complexity and flexibility of the gut related to a species’ capacity for seasonal activity? Cold-water species that remain active during the winter may benefit from a more structurally stable gut that maintains digestive capacity year-round. Conversely, in winter-quiescent warm-water species, the cost of maintaining gastrointestinal tissue may be a substantial contributor to over-winter energy dynamics. We hope to motivate future research that considers the role of the digestive tract in mediating ecophysiological responses to both predictable and unpredictable environmental change.

### 4.1. Caveats and limitations

Though this work involved 14 biweekly sampling events across multiple ponds, sample sizes from individual sampling periods were regularly limited by low catch numbers in Pond C, especially during winter (*n* = 31; table 1). In this population, the dataset was limited to five sampling periods with sufficient replication for analysis. Though dry gut mass data across these five periods were consistent with seasonal patterns seen in the other sampled ponds, caution should be taken when interpreting figures that present data from all sampling periods. Further, repeated lethal sampling of top predators (i.e. pumpkinseed) from these ponds probably increased mortality rates and may have influenced resource availability. Future work should quantify the impact of seasonal resource availability on the phenology and magnitude of gut flexibility. This consideration may be particularly important, as inter-population differences in the timing and flexibility of the GI regions identified here suggest that pond-specific conditions strongly mediate the demand for seasonal gut flexibility.

## Data Availability

All data and code are available online [44]. Supplementary material is available online [45].
